# Contamination of human DNA samples with mouse DNA can lead to false detection of XMRV-like sequences

**DOI:** 10.1186/1742-4690-7-109

**Published:** 2010-12-20

**Authors:** Brendan Oakes, Albert K Tai, Oya Cingöz, Madeleine H Henefield, Susan Levine, John M Coffin, Brigitte T Huber

**Affiliations:** 1 Department of Pathology, Tufts University School of Medicine, 150 Harrison Avenue, Boston, MA 02111, USA; 2 Pharmacology Program, Tufts University School of Medicine, 150 Harrison Avenue, Boston, MA 02111, USA; 3 Department of Molecular Biology and Microbiology, Tufts University School of Medicine, 150 Harrison Avenue, Boston, MA 02111, USA; 4 Genetics Program, Tufts University School of Medicine, 150 Harrison Avenue, Boston, MA 02111, USA; 5 Private Practice, 115 East 72nd Street, New York, NY, USA

## Abstract

**Background:**

In 2006, a novel gammaretrovirus, XMRV (xenotropic murine leukemia virus-related virus), was discovered in some prostate tumors. A more recent study indicated that this infectious retrovirus can be detected in 67% of patients suffering from chronic fatigue syndrome (CFS), but only very few healthy controls (4%). However, several groups have published to date that they could not identify XMRV RNA or DNA sequences in other cohorts of CFS patients, while another group detected murine leukemia virus (MLV)-like sequences in 87% of such patients, but only 7% of healthy controls. Since there is a high degree of similarity between XMRV and abundant endogenous MLV proviruses, it is important to distinguish contaminating mouse sequences from true infections.

**Results:**

DNA from the peripheral blood of 112 CFS patients and 36 healthy controls was tested for XMRV with two different PCR assays. A TaqMan qPCR assay specific for XMRV *pol *sequences was able to detect viral DNA from 2 XMRV-infected cells (~ 10-12 pg DNA) in up to 5 μg of human genomic DNA, but yielded negative results in the test of 600 ng genomic DNA from 100,000 peripheral blood cells of all samples tested. However, positive results were obtained with some of these samples, using a less specific nested PCR assay for a different XMRV sequence. DNA sequencing of the PCR products revealed a wide variety of virus-related sequences, some identical to those found in prostate cancer and CFS patients, others more closely related to known endogenous MLVs. However, all samples that tested positive for XMRV and/or MLV DNA were also positive for the highly abundant intracisternal A-type particle (IAP) long terminal repeat and most were positive for murine mitochondrial cytochrome oxidase sequences. No contamination was observed in any of the negative control samples, containing those with no DNA template, which were included in each assay.

**Conclusions:**

Mouse cells contain upwards of 100 copies each of endogenous MLV DNA. Even much less than one cell's worth of DNA can yield a detectable product using highly sensitive PCR technology. It is, therefore, vital that contamination by mouse DNA be monitored with adequately sensitive assays in all samples tested.

## Background

XMRV (xenotropic murine leukemia virus-related virus) is a novel gammaretrovirus that was identified in 2006 in 10% of prostate cancers [1]. Its functional significance was implied by the recent observation that it is prevalent mainly in more aggressive tumors [2]. In 2009, it was reported that 67% of chronic fatigue syndrome (CFS) patients had this infectious gammaretrovirus, while only a small fraction of healthy volunteers was XMRV-positive [3]. These data were received with enthusiasm because they pointed to a possible infectious etiology of CFS, a chronic disability that is clinically ill-defined. However, several research groups challenged these conclusions almost immediately [4-11] because they could not detect the predicted PCR products or antibodies in cohorts of CFS or prostate cancer patients (reviewed in [12-15]).

Recently, sequences related to other murine leukemia viruses (MLVs) were reported in 80% of CFS patients versus only a small percentage of healthy controls [16]. This finding implicated different retroviruses specifically linked to this patient population than the originally described XMRV [3]. The similarity of such sequences to large numbers of endogenous MLVs present in any mouse strain [17-19] complicates interpretation of detection of such sequences in clinical studies since possible contamination of the human samples with mouse DNA [14,20] has to be rigorously ruled out to validate such results.

Our laboratory has been involved in CFS research since 2005 and has a substantial library of samples stored from a cohort of patients and controls. Using a nested PCR for XMRV, we detected one XMRV-like and various MLV-like sequences, but also observed a 100% correlation between samples that were positive for XMRV/MLV sequences and those positive for mouse DNA, while most samples negative for XMRV/MLV were also negative for mouse DNA. These results imply frequent laboratory contamination with minute and highly variable quantities of mouse DNA.

## Results

### Study populations

We analyzed a library of 111 stored DNA samples that had been collected from the peripheral blood mononuclear cells (PBMC) of CFS patients in 2005 for an unrelated project (see Methods section for description). In addition, we collected 37 blood samples (one CFS and 36 healthy controls) in 2009-2010.

### TaqMan qPCR specific for XMRV did not reveal positive individuals

**Figure 1:**
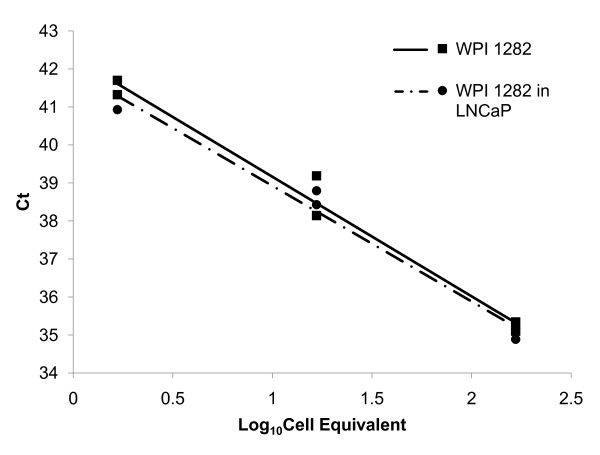
**Sensitivity of TaqMan qPCR for IN region in XMRV *pol***. Titration of DNA from WPI-1282 (1.7, 16.7, and 166.7 cell equivalents) in the absence (square, solid line, slope = -3.14) or in the presence of 8.3 × 10^5 ^cell equivalents of genomic LNCaP DNA (circle, dotted line, slope = -3.04). 1.7 cell equivalents of WPI-1282 genomic DNA is detectable in 8.3 × 10^5 ^cell equivalents of background DNA. Samples were run in duplicates. All qPCR reactions were run for 60 cycles. Samples that did not produce a signal after 60 cycles were assumed negative for XMRV. Ct = Cycle Threshold

The original XMRV results from patients with prostate cancer and CFS were obtained using a sensitive nested PCR assay for XMRV [1,3] that also detects endogenous MLV sequences in murine genomic DNA. These data were later extended, employing a qPCR assay specific for a region in the XMRV *pol *gene not cross-reactive with any sequence known to be present in mouse DNA [2, Singh, personal communication]. To test our cohort for the presence of XMRV sequences, we analyzed PBMC DNA with this 2^nd ^qPCR assay, using the primers and probe as described in [2]. Titration of DNA from an XMRV-positive lymphoblastoid cell line, WPI-1282 (kindly provided by the Whittemore Peterson Institute (WPI)), resulted in detection of XMRV down to 10-12 pg, equivalent to two cells, in the presence or absence of 5 μg control DNA isolated from the human LnCaP cell line (Figure 1). However, no positive response (C_t _> 60) was obtained with DNA from 112 CFS patients and 36 healthy controls, when tested at 600 ng to 5 μg per reaction (data not shown). These data indicated that our samples were either XMRV-negative or had more divergent MLV sequences than originally described [1,3]. In the latter case, the qPCR assay used, which is sensitive to small sequence differences, would not have allowed detection.

### Nested PCR for XMRV *gag *yielded a high frequency of positive samples

**Figure 2:**
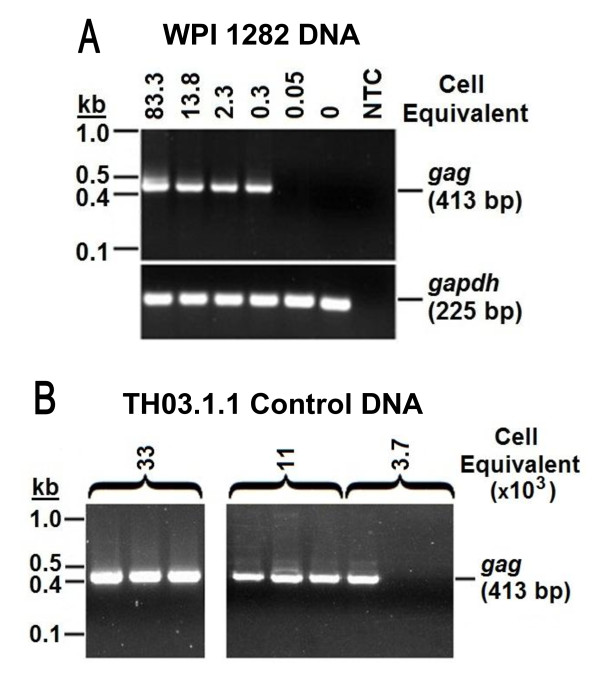
**Sensitivity of nested PCR for XMRV *gag***. A) Titration of genomic DNA from WPI-1282. PCR amplicons from 83.3, 13.8, 2.3, 0.3, 0.05 and 0 cell equivalents of genomic DNA from the WPI-1282 cell line in the presence of 3.3 × 10^4 ^cell equivalents of LNCaP genomic DNA were run on a 1.5% agarose gel to show the sensitivity of the assay. *gapdh *was used as the loading control. XMRV *gag *yields an expected product of 413 bp. NTC = No Template Control. B) Representative example of nested PCR for XMRV *gag*. Sample TH03.1.1 was first tested at 3.3 × 10^4 ^cell equivalents of genomic DNA, followed by limiting dilutions of 1.1 × 10^4 ^and 3.7 × 10^3 ^cell equivalents. Once a dilution had 1 out of 3 samples positive for *gag*, the positive band was purified and sequenced.

To explore the possibility that XMRV sequences in humans are more divergent than previously reported, we used the nested PCR assay for XMRV *gag *sequences mentioned above, which also detects many endogenous MLV proviruses, as described [1]. A preliminary titration experiment revealed that MLV-like sequences could be detected in 2-3 pg of WPI-1282 DNA, equivalent to <1 cell, when mixed with 200 ng control DNA (see above) (Figure 2). This assay was used to test DNA in triplicates of 200 ng each from our CFS and control cohorts. Surprisingly, a high proportion of DNA samples from the healthy volunteers (19/36), but only 2/112 of the CFS patients, yielded PCR products of the correct size, as tested on an agarose gel. None of the "no template" control samples, included in each assay at least in triplicate, gave positive results. These data suggested that XMRV-related viruses may be highly prevalent in the human population, but no special link of these viruses to CFS patients was indicated. While all the blood samples were processed in the Huber laboratory, it should be noted that the CFS cohort mainly consisted of banked samples collected and processed in 2005, whereas the healthy volunteers were recruited more recently, between November of 2009 and May of 2010, and, as discussed later, were processed using a slightly different protocol.

### Sequence analysis of the *gag *PCR products revealed high polymorphism

**Figure 3:**
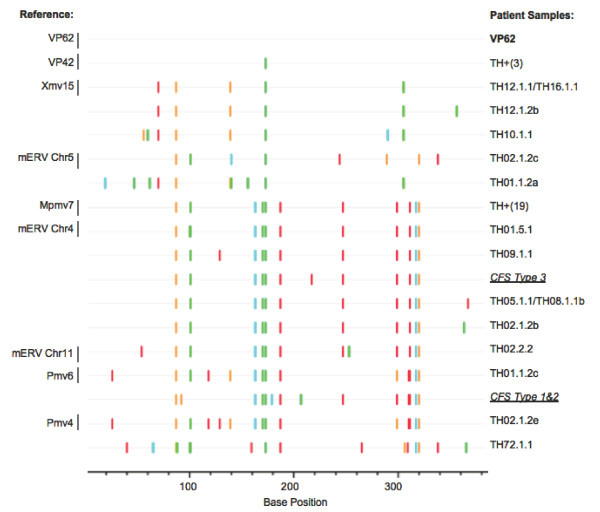
***Gag *sequences from patient samples**. Individual 382 bp sequences, free of double peaks and confirmed through forward and reverse sequencing, are compared in a Highlighter plot to the control WPI-1282 cell line sequence, VP62. The samples were coded to remain anonymous, with the first number being the patient number, the second number being the bleed number, the third number being the tube of DNA, and the letter showing that we have multiple sequences in the same tube of DNA. Identical sequences were collapsed into individual clusters, those with more than two sequences are labeled TH+(N), where N is the total number of sequences in that cluster. CFS Type 1, 2 & 3 are from Lo *et al. *[15]. Each vertical line shows a single nucleotide difference between the labeled sequence and the control VP62 sequence.

To determine the relationship among the various PCR products, we obtained their DNA sequences. We observed that most amplicons contained mixtures of sequences, thus, necessitating limiting dilutions of the original DNA samples to obtain pure sequences for analysis (Figures 2B &3, Additional File 1; Table S1). A total of 37 clean sequences of single PCR products (designated TH for "Tufts Huber") were obtained in this way from 21 samples (19 healthy controls and 2 CFS). Surprisingly, a high degree of diversity was seen in these viral sequences (Figure 3 Additional File 1; Table S1), revealing both XMRV-like and endogenous MLV sequences and implying 15 different virus strains. While 3 healthy controls had sequences that were identical to the corresponding segment of XMRV strain VP42, a viral isolate that was originally found in prostate cancer [1] and later in CFS patients [3], the remaining samples were either identical or closely related to known endogenous MLVs [17-19].

**Figure 4:**
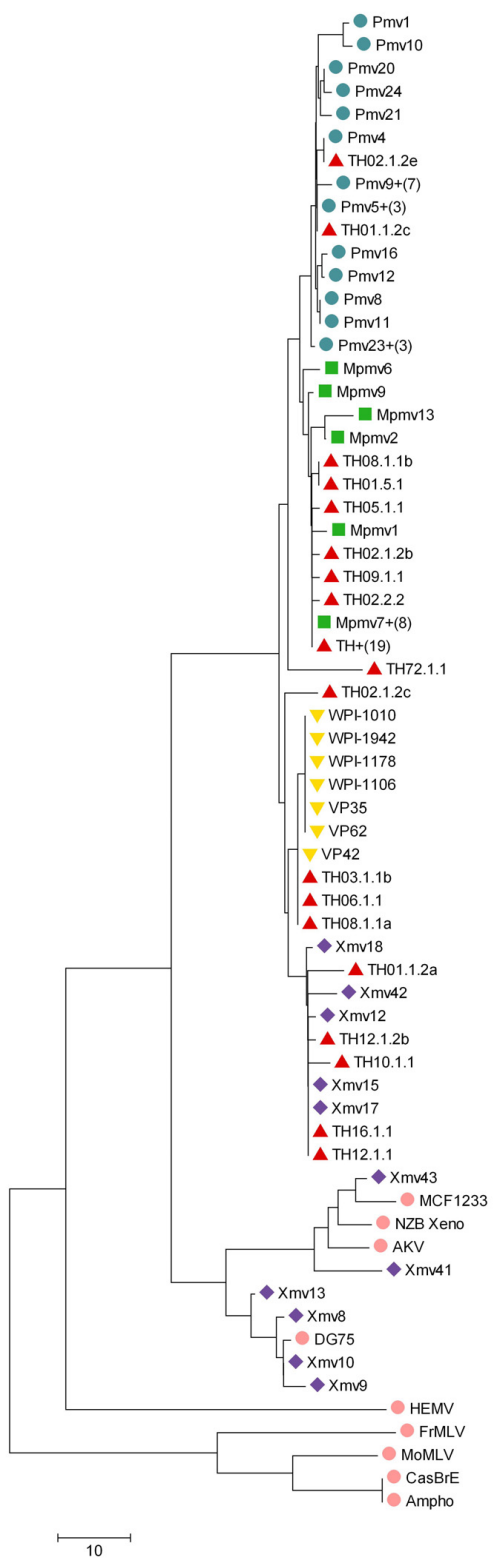
**Neighbor Joining Tree of *gag *fragments**. A neighbor-joining tree was constructed using the 382 bp *gag *fragments detected from the PBMC DNA of 17 healthy controls and 2 CFS patients, along with various endogenous and exogenous MLV sequences. Identical sequences were collapsed into individual clusters, where a representative member is shown followed by "+(N)", where N is the total number of sequences in that cluster. Distances were calculated based on the absolute number of base substitutions; all sites containing gaps were ignored. Note the extensive variation of sequences detected in our samples (TH, shown in red), which cluster with known Xmv (purple), Pmv (blue), Mpmv (green) and XMRV (yellow) sequences.

The sequences obtained were also analyzed by constructing neighbor-joining trees (Figure 4). Again, our data indicate a high degree of polymorphism in the MLV-like sequences found. In contrast to the published VP [1] and WPI [3] XMRV sequences, which are tightly clustered, the *gag *sequences found in this study were dispersed, similar to the sequences reported in [16]; *i.e*., the 15 unique XMRV-related partial *gag *sequences found among from the 37 single PCR products were distributed over a minimum of 3 clusters, each of which contains endogenous MLV sequences of a different subtype (XMV, PMV, and MPMV (xenotropic, polytropic, modified polytropic MLV)).

### Tests for mouse DNA contamination revealed correlation with viral sequences

**Figure 5:**
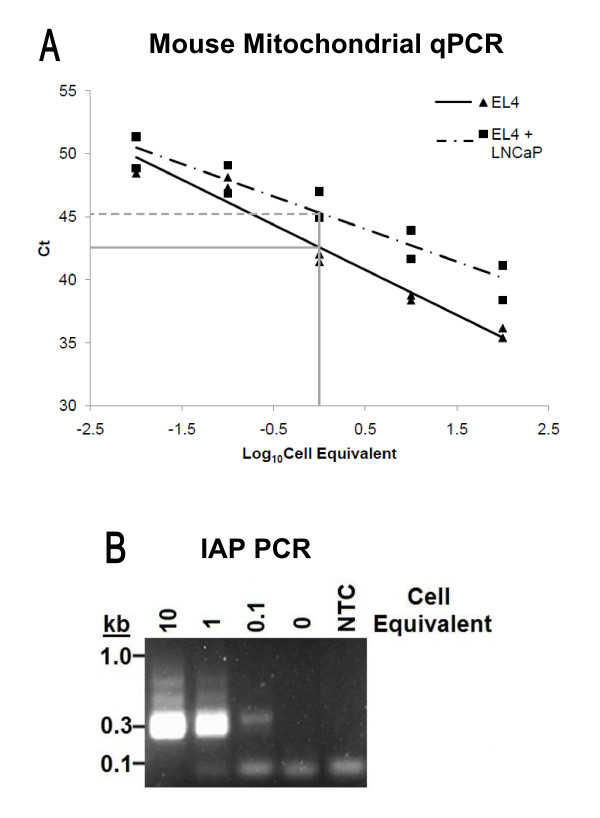
**Tests for mouse DNA**. A) TaqMan qPCR for murine mitochondrial cytochrome oxidase (*mcox2)*. Titration of DNA from the murine EL4 cell line (100, 10, 1, 0.1, and 0.01 cell equivalents) in the absence (triangle, solid line, slope = -3.58) or in the presence of 3.3 × 10^4 ^cell equivalents of background genomic LNCaP DNA (square, dotted line, slope = -2.58). 0.1 cell equivalents of murine DNA were observed in 3.3 × 10^4 ^cell equivalents of background DNA. Samples were run in duplicate. All qPCR reactions were run for 60 cycles. Samples that did not produce a signal after 60 cycles were considered negative for murine DNA. B) IAP PCR. Titration of DNA from the murine EL4 cell line (10, 1, and 0.1 and 0 cell equivalents) in the presence of 3.3 × 10^4 ^cell equivalents of genomic LNCaP DNA. The limit of detection was 0.1 cell equivalents of murine DNA in 3.3 × 10^4 ^cell equivalents of background DNA. Although not visible here, bands of different sizes and unrelated sequence are sometimes visible in samples analyzed with human DNA alone. NTC = No Template Control.

Endogenous MLVs are present in high copy number in all inbred and many species of wild mice, making mouse DNA a possible source of the sequences observed. To test whether contamination with mouse DNA might account for the observed results, all human DNA samples were screened using two different assay systems, a TaqMan qPCR assay for murine mitochondrial cytochrome oxidase, c*ox2 *(W. Switzer, personal communication) and a single PCR assay for the highly abundant intracisternal A-type particle (IAP) long terminal repeat sequences, developed by us (OC and JMC, in preparation) (see DNA sequences of some IAP amplicons in Additional File 2; Figure S1). Both assays had similar sensitivity, detecting the target sequences in 0.6 pg of mouse DNA, equivalent to 1/10 of a cell in a background of 200 ng LnCap DNA (Figure 5A &5B). Using these two test systems, we observed that many samples, both CFS and control, were positive for these types of sequence, while all "no template" controls were negative. A direct comparison of the *gag *PCR results with those obtained in the two assays for mouse DNA revealed a 100% correlation between samples positive for the former and mouse DNA; all human DNA samples that were positive in the *gag *PCR assay were also positive for IAP sequences, and all but 2 were positive for mouse *cox2 *sequences (Table 1). In addition, nearly half (62/127) of the samples were positive for mouse DNA by either IAP or both assays, but did not yield a detectable MLV signal. These findings are in agreement with our observation that the two PCR assays for mouse DNA are at least 10-fold more sensitive than the XMRV *gag *PCR assay, when tested on genomic mouse DNA, and that the IAP assay is more sensitive than the *cox2 *assay for detection of mouse DNA. Overall, our data are consistent with the conclusion that the positive results obtained with the XMRV *gag *PCR assay are due to variable contamination of the human samples with mouse DNA, most likely in laboratory reagents.

**Table 1:** Correlation of MLV DNA sequence detection with mouse DNA contamination

			CFS Patients		Healthy Controls**	
XMRV GAG	*cox*	IAP	# of Samples (n = 112)	Percent	# of Samples (n = 36)	Percent
**+**	**+**	**+**	2*	1.8	17	47.2

**-**	**-**	**-**	53	47.3	12	33.3

**+**	**-**	**-**	0	0	0	0

**+**	**-**	**+**	0	0	2	5.6

**+**	**+**	**-**	0	0	0	0

**-**	**+**	**+**	10	9.0	1	2.8

**-**	**-**	**+**	47	42.0	4	11.1

**-**	**+**	**-**	0	0	0	0

*One CFS sample from 2005 collection, and one CFS sample from 2010 collection. All the other CFS samples were collected in 2005.

**All collected in 2009-2010.

## Discussion

In 2005, we initiated a study to examine the expression level of an endogenous human betaretrovirus, HERV-K18, in chronically ill CFS patients versus healthy controls. For this purpose, we accumulated a library of DNA samples from CFS patients which has allowed us to investigate the possible association of XMRV with this disease [3]. We initiated our studies on XMRV using a TaqMan qPCR assay for a region in XMRV *pol *that is unique to XMRV and does not detect any sequences in genomic DNA from laboratory strains of inbred mice [2]. None of the samples from either CFS patients or healthy controls was positive in this assay, although we were able to detect a signal from two XMRV-infected lymphoblastoid cells (cell line WPI-1282) in a background of DNA from up to 10^6 ^human LnCaP cells. In our hands, the qPCR assay is 10-fold less sensitive than the nested XMRV *gag *PCR assay when tested on the same XMRV-positive cell line, since the latter can detect a signal in DNA from <1 cell. This difference is a consideration for the negative results we obtained as the sensitivity of the qPCR assay may not have been adequate for the detection of minute amounts of XMRV. We are not aware of any other group who has used this technique for the detection of XMRV in the DNA of freshly isolated PBMC. However, Danielson *et al*. recently reported that they could only detect XMRV sequences, using XMRV *env*, but not *gag*, primers [21].

In contrast to the qPCR results, we were able to readily detect XMRV using the nested PCR originally described by Urisman *et al. *[1], and we found many more positive samples in our healthy control cohort, compared to the CFS cohort. Of possible relevance for the interpretation of these findings may be the fact that the samples from the two cohorts were prepared years apart, although all in the same laboratory using somewhat different protocols and reagents. It is also important to point out that individual DNA samples remained reproducibly positive or negative on repeat examination rendering the possibility of random contamination of the PCR assays very unlikely. Furthermore, each assay contained positive and negative controls which were 100% correlative; *i.e*., the DNA from the XMRV-infected cell line was always positive and the no-template control or LnCaP DNA was always negative. Thus, it is unlikely that contamination occurred at the time of setting up the PCR reactions.

To further understand the origin of the positive PCR signals, we determined the DNA sequences of the *gag *PCR products. In most cases, it was only possible to obtain unique sequences from PCR products after dilution of the input DNA to an extent where single molecules were amplified, since initial studies showed that most of the positive samples contained mixtures of closely related sequences. In this way, we obtained 15 different sequences from a total of 37 single PCR products. When compared to the collection of endogenous MLV sequences extracted from the sequenced mouse genome [18,22], these sequences included examples from all parts (XMV, PMV, and MPMV) of the resulting neighbor-joining tree, as well as a cluster of three sequences identical (in this region) to the VP42 isolate of XMRV. With regard to the latter result, it is of significance that no VP42 plasmid, nor VP42-containing cell line, nor isolated DNA, was present in the Huber laboratory that could have resulted in contamination (WPI-1282 contains VP62 which differs by one base change in the region analyzed). The genomic DNA from the three healthy volunteers who had XMRV VP42 sequences also contained other MLV sequences. Thus, it is not possible for us to distinguish which one of the retroviruses stemmed from mouse DNA contamination; *i.e.*, it is formally possible that VP42 is an actual human retrovirus. It is also possible that it is an endogenous provirus, not present in the sequenced C57Bl/6 genome, but present in the mouse species responsible for the sequences observed [19]. In the former case, the presence of VP42 in DNA from healthy control samples, but not CFS patients, would indicate that this virus is spread randomly through the human population, with no particular link to CFS. Further analyses are required to clarify this issue.

The presence of mixtures of MLV sequences, all closely related to known endogenous MLVs [17-19], in many of the DNA samples tested is not easily reconciled with infection of human hosts with the corresponding viruses (reviewed in [14,20]). Two assays specific for murine DNA, for mitochondrial *cox2 *and IAP sequences, were used to test the possibility that there might be trace amounts of mouse DNA contaminating some of the samples. Consistent with this idea, we found that each DNA sample that was positive for XMRV/MLV also was positive for mouse DNA by the IAP assay, while >50% of XMRV/MLV-negative samples were positive for mouse DNA which is particularly striking in the CFS group. Again, these results were confirmed in repeat experiments and never deviated in subsequent analyses, suggesting that contamination happened either during collection of blood, isolation of PBMC, or during the preparation of the DNA from the PBMC. We interpret these data that possible contamination with mouse DNA is ubiquitous, but the level seemed to vary significantly from batch to batch of sample preps, although all experimental procedures were carried out in the same facility. In particular, although samples collected at both times showed signs of contamination, the level of contamination in the normal controls collected in 2009-2010 was noticeably greater than in the CFS samples from 2005. To date, we have not been able to pinpoint a specific reagent or laboratory vessel for being consistently positive for mouse DNA, but preliminary experiments implicate both fetal calf serum (FCS) and phosphate buffered saline (PBS), although large variations in the surmised amount of contaminating mouse DNA were observed from bottle to bottle. All blood samples were collected in heparin tubes rendering the anti-coagulant also a likely suspect for mouse DNA contamination. However, a comparison of parallel blood collections from the same healthy individual in heparin, Na-citrate and EDTA tubes did not support this hypothesis. In this particular set of samples only one DNA aliquot from Na-citrate-collected blood was positive for mouse DNA (results not shown).

Currently there are highly discordant reports in the literature about the prevalence of XMRV in CFS and prostate cancer patients (reviewed in [12-15]). The original publication on CFS patients reported that almost 70% of these patients, but less than 5% of healthy individuals, harbor this virus [3], and that infectious virus and antiviral antibodies could be detected in blood from these patients. Several reports have appeared in the literature since then contesting these findings [4-6,8,9], while a recent publication claimed that 80% of CFS patients, but not healthy controls, contained endogenous MLV-like sequences, but were negative for mouse mitochondrial DNA [16]. The sequences from CFS patients identified in this latter paper were distinct from the XMRV of the original reports. A plausible explanation for these discrepant results has not been put forward to date [13,14], but it is worth pointing out that the sequences identified in the latter report were similar to the ones we found in the present study. Endogenous MLVs are abundant in all laboratory mouse strains [17,18], as well as in wild *Mus *species [19] and are carried by some human cell lines that have been propagated *in vivo *in nude mice [20]. Thus, extreme precautions have to be taken to exclude contamination with mouse DNA or DNA from any abundant MLV-producing cell line.

## Conclusions

In our study we have observed that 100% of human DNA samples prepared in our laboratory that were positive for XMRV/MLV sequences were also positive for minute quantities of mouse DNA. Together with the similarity of the MLV sequences to multiple identified endogenous MLVs [17-19], this result provides a strong suspicion that the viral sequences detected in these samples were actually of murine origin. It is important to point out that negative controls included in each assay never yielded positive results, either for XMRV/MLV, or for mouse DNA, excluding the possibility that contamination with mouse DNA occurred at the bench during the final PCR assay, even though mouse derived cells and tissues are regularly used in our laboratory. Of particular interest is the wide variety of sequences that we obtained, spanning both XMRV and various MLV sequences. While most of the MLV-related sequences were identical to *gag *segments in nonecotropic MLVs from inbred mice [17,18], some were found to be unique; *i.e*., they have so far not been identified in the sequenced mouse genome [22], but may be present in other laboratory strains or wild mice. Thus, our data are compatible with the conclusion that the detection of MLV-related sequences in human genomic DNA samples could be due to contamination with minute and variable quantities of mouse DNA, most likely contained in various laboratory reagents.

## Methods

### Sample collection

All samples were collected according to the institutional guidelines of Tufts University, after receiving informed consent. The 36 healthy individuals (15 females and 21 males) were recruited on a voluntary basis by the Huber laboratory and were between 18 and 65 years of age. The 112 CFS patients (89 females, 20 males and 3 unknown), recruited by Dr. Susan Levine, were between 18 and 65 years of age and resided in the Northeastern United States. All patients were diagnosed for CFS according to CDC criteria [23], and the majority was completely disabled. The cohort comprised a combination of those with an abrupt and others with a gradual onset of symptoms.

### Preparation of human blood samples

Approximately 30 ml of blood were drawn into three heparinized tubes (Becton Dickinson) and shipped overnight (CFS patients) or processed immediately (healthy controls). The blood collection tubes from each individual were consolidated into one 50 ml tube and diluted with PBS, containing CaCl_2 _and MgCl_2 _(Sigma) at a 1:1 ratio. 15 ml of Ficoll (GE Healthcare) was added to two new 50 ml tubes, and 25 ml of the diluted blood was gently layered on top of the Ficoll, followed by a 30 min centrifugation in a Sorvall RT7plus rotor at 2000 rpm at room temperature and collection of PBMCs from the interface. 10 ml of plasma were also collected from each sample and stored at -80°C. The collected PBMCs were diluted with PBS (2005 collection) or RPMI-1640 Medium (Sigma), supplemented with 10% FCS (Gemini BioProducts), 100 U/ml penicillin (Sigma), 0.1 mg/ml streptomycin (Sigma), 2 mM L-glutamine (Sigma), and 1 mM sodium pyruvate (Sigma) (2010 collection) (2009-2010 collection) (complete RPMI) at a 1:1 ratio and then pelleted at 2000 rpm for 5 min. The supernatant was aspirated, and the pellet of PBMCs was resuspended in 20 ml of PBS (2005 collection) or complete RPMI (2009-2010 collection). Cells were counted using a light microscope and a hemocytometer, aliquoted to 5 × 10^6 ^cells per tube, spun down and resuspended in 350 μl of Buffer RLT Plus (Qiagen) (1% β-mercaptoethanol). Samples were stored in this lysis buffer at -80°C.

### DNA isolation from PBMCs

DNA was isolated using the procedures provided by the AllPrep DNA/RNA Mini Kit (Qiagen). Briefly, 350 μl of PBMC lysate (RLT buffer, see above) (5 × 10^6 ^cells) were placed on the DNA spin column, which was centrifuged at 10,000 rpm for 30 s in an Eppendorf 5417C Centrifuge. The column was then transferred to a new collection tube. 500 μl AW1 Buffer (Qiagen) was added to the column, followed by a 15 s spin at 10,000 rpm. The flow-through was discarded, and the column was transferred to a new collection tube. 500 μl of AW2 Buffer (Qiagen) was added to the column, followed by a 2 minute centrifugation at full speed. The flow-through was discarded, and the column was transferred to a new 1.5 ml collection tube. 100 μl of Buffer EB (Qiagen) was added directly to the column, followed by 1 minute incubation at room temperature. Finally, the column was centrifuged at 10,000 rpm for 1 min to elute DNA. DNA concentration was determined using 1 μl of sample on a Thermo Scientific Nanodrop 2000 Spectrophotometer.

### TaqMan qPCR assay for XMRV *pol*

Primers and probe, as designed by Schlaberg *et al. *[2], were ordered from Applied Biosystems (see Table 2 for sequences). The reaction mix for the TaqMan qPCRs contained 1× Gene Expression Master Mix (Applied Biosystems), 900 nM forward and reverse primers, 250 nM probe, and 200 ng of DNA in a reaction volume of 20 μl. The assay was validated with DNA from the WPI-1282 cell line containing VP62 XMRV (kindly supplied by J. Mikovits, WPI). The same DNA served as positive control in each assay, which also included a no-template negative control. Thermocycler conditions were 95°C for 10 minutes, followed by 60 cycles of 95°C for 15 s and then 60°C for 1 minute, using 96-well Optical Reaction Plates (Applied Biosystems) on a 7300 Real Time PCR System by Applied Biosystems. All reactions were performed in triplicate. Quality of DNA was assessed using a TaqMan qPCR for the ribosomal 18 S gene in the same reaction (Applied Biosystems).

**Table 2:** Primers and probes used for TaqMan qPCRs, primary PCRs, and nested PCRs.

Primer	Sequence
XMRV4552F	5'-CGA GAG GCA GCC ATG AAG G-3'
XMRV4673R	5'-CCC AGT TCC CGT AGT CTT TTG AG-3'
XMRV4572MGB	5'-6FAM-AGT TCT AGA AAC CTC TAC ACT C-MGBNFQ-3'
GAG-O-F	5'-CGC GTC TGA TTT GTT TTG TT-3'
GAG-O-R	5'-CCG CCT CTT CTT CAT TGT TC-3'
GAG-I-F	5'-TCT CGA GAT CAT GGG ACA GA-3'
GAG-I-R	5'-AGA GGG TAA GGG CAG GGT AA-3'
MCox2-F2	5'-TTC TAC CAG CTG TAA TCC TTA-3'
MCox2-R1	5'-GTT TTA GGT CGT TTG TTG GGA T-3'
MCox2-PR1	5'-FAM-CGT AGC TTC AGT ATC ATT GGT GCC CTA TGG T-MGBNFQ-3'
MCox2-P1	5'-FAM-TTG CTC TCC CCT CTC TAC GCA TTC TA-MGBNFQ-3'
IAP-Forward	5'-ATA ATC TGC GCA TGA GCC AAG G-3'
IAP-Reverse	5'-AGG AAG AAC ACC ACA GAC CAG A-3'

### Nested PCR assay for XMRV *gag*

Identical primers as originally described by Urisman *et al. *[1] and also employed by the Mikovits group [3] were used. The reaction mix for all PCRs consisted of 1× HotStart-IT™FideliTaq™Master Mix, 200 nM forward and reverse primers, and 200 ng of sample DNA in a 50 μl reaction volume. The WPI-1282 lymphoblastoid cell line was used as a positive control [3]. Thermocycler conditions for the first PCR were 2 minutes at 94°C, followed by 30 cycles of 94°C for 30 s, 58°C for 30 s, and 72°C for 45 s and then finished off with 72°C for 7 minutes. Once the first PCR was complete, 2 μl of DNA from the first PCR was used for the second PCR. The second PCR consisted of 1× HotStart-IT™FideliTaq™Master Mix, 200 nM forward and reverse primers, and 200 ng of sample DNA in a 50 μl reaction volume. Thermocycler conditions for the second PCR were 2 minutes at 94°C, followed by 30 cycles of 94°C for 30 s, 60°C for 30 s, and 72°C for 30 s and then finished off with 72°C for 7 minutes. Once the second PCR was complete, 15 μl of the samples were run on a 1.5% agarose gel for 1 h at 100 volts. Images of gels were taken using a VersaDoc Imaging System (Biorad). The expected fragment size of the second PCR is 413 bp [1].

All positive samples from the second XMRV nested PCR were isolated using a Qiaquick PCR Purification Kit (Qiagen). DNA sequencing was performed by the Tufts University Core Facility. Once sequenced, the traces were monitored for double peaks, and sequences with double peaks were discarded. Samples that had mixed sequences were diluted, and the nested PCR was repeated. Only clean sequences with the forward sequence matching the reverse sequence were used for phylogenetic analysis.

### TaqMan qPCR assay for mouse mitochondrial *cox2*

Sequences for primers and probes were kindly supplied by Dr. Switzer, CDC (Personal Communication) (see Table 2). Primers and Probes were ordered from Applied Biosystems. The reaction mix contained 1× Gene Expression Master Mix (Applied Biosystems), 900 nM forward and reverse primers, 250 nM probe, and 200 ng of DNA in a reaction volume of 20 μl. DNA isolated from the murine EL4 cell line, diluted in 200 ng of human LNCaP DNA, was used as a positive control. Thermocycler conditions were 95°C for 9 minutes, followed by 60 cycles of 95°C for 30 s and 62°C for 30 s. 96-well plates were used on a 7300 Real Time PCR System by Applied Biosystems. All reactions were performed in duplicate or triplicate. Quality of DNA was assessed using a TaqMan qPCR for the ribosomal 18 S gene in the same reaction (Applied Biosystems).

### PCR assay for Mouse IAP sequences

Primers were designed by the Coffin Laboratory (OC and JMC, in preparation) and ordered from Invitrogen. The reaction mix for all PCRs consisted of 1× HotStart-IT™FideliTaq™Master Mix, 1 μM forward and reverse primers, and 200 ng of sample DNA in a 50 μl reaction volume. DNA isolated from the murine EL4 cell line was diluted into 200 ng of human DNA (LNCaP) and used as a positive control. Thermocycler conditions were 94°C for 2 minutes, followed by 45 cycles of 94°C for 30 s, 58°C for 30 s, and 72°C for 20 s and then finished off with 72°C for 7 minutes. Samples were then run on a 1.5% agarose gel with sequence lengths varying between 200 and 300 bp. Images of gels were taken using a VersaDoc Imaging System (Biorad). IAP PCR products were cloned and sequenced and yielded the expected results (see Additional File 2; Figure S1).

## List of abbreviations

CFS: Chronic Fatigue Syndrome; FCS: fetal calf serum; IAP: intracisternal A-type particle; MLV: murine leukemia virus; MPLV: modified polytropic MLV; PBMC: peripheral blood mononuclear cells; PBS: phosphate buffered saline; PMV; polytropic MLV; WPI: Whittemore Peterson Institute; XMRV: xenotropic murine leukemia virus-related virus; XMV: xenotropic MLV.

## Competing interests

The authors declare that they have no competing interests.

## Authors' contributions

BTH, AKT and BO conceived and designed the study. AKT, BO and MHH carried out the experiments. SL collected samples from the CFS patient cohort. AKT, BO, MHH, OC and JMC analyzed the data. BTH drafted the manuscript. All authors read and approved the final manuscript.

## Supplementary Material

Additional File 1 **Supplementary Table 1 - List of identical sequences grouped into clusters for analysis**. Each cluster contains fragments that are identical in the corresponding 382 bp *gag *region. Click here for file

Additional File 2 **Supplemental Figure 1 - IAP sequences**. IAP sequences amplified from the indicated control human DNA samples using the primers shown in Table II were cloned into a TOPO vector and sequenced. Four representative sequences are shown. Each sequence had a 100% match in the sequenced mouse genome. Adenine (A) = Green, Cytosine (C) = Blue, Guanine (G) = Black, Thymine (T) = Red. Click here for file
