# Supplementary material for: Contamination of human DNA samples with mouse DNA can lead to false detection of XMRV-like sequences
Source: Retrovirology. 2010 Dec 20;7:109. doi: 10.1186/1742-4690-7-109 (PMC3022687; doi:10.1186/1742-4690-7-109)
Supplement: Additional File 1 — Supplementary Table 1 - List of identical sequences grouped into clusters for analysis. Each cluster contains fragments that are identical in the corresponding 382 bp gag region. [file 1742-4690-7-109-S1.DOC]

**Table S1. List of identical sequences grouped into clusters for analysis**

| **Cluster Designation** | **Sample ID** |  | **Cluster Designation** | **Provirus** |
| --- | --- | --- | --- | --- |
|  |  |  |  |  |
| TH+(19) | TH01.1.2b |  | Mpmv7+(8) | Mpmv3 |
|  | TH01.7.1a |  |  | Mpmv4 |
|  | TH01.7.1b |  |  | Mpmv5 |
|  | TH01.7.1c |  |  | Mpmv7 |
|  | TH01.7.1d |  |  | Mpmv8 |
|  | TH01.8.1 |  |  | Mpmv10 |
|  | TH02.1.2a |  |  | Mpmv11 |
|  | TH02.1.2d |  |  | Mpmv12 |
|  | TH02.2.1 |  |  |  |
|  | TH03.1.1a |  | Pmv9+(7) | Pmv2 |
|  | TH03.1.1c |  |  | Pmv5 |
|  | TH04.1.1 |  |  | Pmv7 |
|  | TH06.1.2 |  |  | Pmv9 |
|  | TH07.1.1 |  |  | Pmv13 |
|  | TH11.1.1 |  |  | Pmv15 |
|  | TH12.1.2a |  |  | Pmv17 |
|  | TH17.1.1 |  |  | Pmv22 |
|  | TH20.1.1 |  |  |  |
|  | TH21.1.1 |  | Pmv5+(3) | Pmv5 |
|  |  |  |  | Pmv6 |
| TH+(3) | TH03.1.1b |  |  | Pmv18 |
|  | TH06.1.1 |  |  |  |
|  | TH08.1.1a |  | Pmv23+(3) | Pmv14 |
|  |  |  |  | Pmv19 |
| TH12.1.1/TH16.1.1 | TH12.1.1 |  |  | Pmv23 |
|  | TH16.1.1 |  |  |  |
|  |  |  |  |  |
| TH05.1.1/TH08.1.1b | TH05.1.1 |  |  |  |
|  | TH08.1.1b |  |  |  |
|  |  |  |  |  |
| None | TH01.1.2a |  |  |  |
|  | TH01.1.2c |  |  |  |
|  | TH01.5.1 |  |  |  |
|  | TH02.1.2b |  |  |  |
|  | TH02.1.2c |  |  |  |
|  | TH02.1.2e |  |  |  |
|  | TH02.2.2 |  |  |  |
|  | TH09.1.1 |  |  |  |
|  | TH10.1.1 |  |  |  |
|  | TH12.1.2b |  |  |  |
|  | TH72.1.1 |  |  |  |
