# Supplementary material for: Contamination of human DNA samples with mouse DNA can lead to false detection of XMRV-like sequences
Source: Retrovirology. 2010 Dec 20;7:109. doi: 10.1186/1742-4690-7-109 (PMC3022687; doi:10.1186/1742-4690-7-109)
Supplement: Additional File 2 — Supplemental Figure 1 - IAP sequences. IAP sequences amplified from the indicated control human DNA samples using the primers shown in Table II were cloned into a TOPO vector and sequenced. Four representative sequences are shown. Each sequence had a 100% match in the sequenced mouse genome. Adenine (A) = Green, Cytosine (C) = Blue, Guanine (G) = Black, Thymine (T) = Red. [file 1742-4690-7-109-S2.PDF]

```
                      10        20        30        40        50        60        70
              ....|....|....|....|....|....|....|....|....|....|....|....|....|....|
TH1.7-IAP3    GCTCTGCCTTCCCCGTGACGTCAACTCGGCCGATGGGCTGCAGCCAATCAGGGAGTGACACGTCCGAGGC
TH11.1-IAP4   --...............................................................T....
TH1.7-IAP6    -.....................................................................
TH1.7-IAP7    -----------------------...............................................

                      80        90       100       110       120       130       140
              ....|....|....|....|....|....|....|....|....|....|....|....|....|....|
TH1.7-IAP3    GAAGGAGAATGCTCCTTAAGAGGGACGGGGTTTCGTTTTCTCTCGCTCTTGCTTCTTGCTCTCTTGCTTC
TH11.1-IAP4   ......T...T........T........................--------------------------
TH1.7-IAP6    ........................................G...---------------...........
TH1.7-IAP7    ............................................---------------...........

                     150       160       170       180
              ....|....|....|....|....|....|....|....|....|.
TH1.7-IAP3    TTGCTCTCTTGCTTCCTGCACCCTGGCTCCTGAAGATGTAAGAAA-
TH11.1-IAP4   ----...........T.A...T..T.................C..T
TH1.7-IAP6    ......................T.....................--
TH1.7-IAP7    .............................................-
```
